# Investigation on the Cooperative Grasping Capabilities of Human Thumb and Index Finger

**DOI:** 10.3389/fnbot.2019.00092

**Published:** 2019-11-05

**Authors:** Xiaojing Chen, Zhiguo Li, Yuqing Wang, Jizhan Liu, Dezong Zhao

**Affiliations:** ^1^College of Mechanical and Electronic Engineering, Northwest A&F University, Yangling, China; ^2^School of Mechanical and Power Engineering, Henan Polytechnic University, Jiaozuo, China; ^3^School of Agricultural Equipment Engineering, Jiangsu University, Zhenjiang, China; ^4^Department of Aeronautical and Automotive Engineering, Loughborough University, Loughborough, United Kingdom

**Keywords:** thumb and index finger, object mass and diameter, human characteristics, cooperative grasping capabilities, robotic hand

## Abstract

The maximum cooperative grasping mass and diameter of the human thumb and index finger were investigated by 7560 grasp-release trials on various masses of solid cylinders and various sizes of rings. The maximum grasping mass of the participants’ thumb-index finger depended on gender, age and the sum of thumb-index finger lengths (*P* < 0.05), but not on the hand-used and ratio of index finger to thumb length (*P* > 0.05). The maximum grasping diameter of the participants’ thumb-index finger depended on the age, sum of thumb-index finger lengths and ratio of index finger to thumb length (*P* < 0.05), but not on the gender and hand-used (*P* > 0.05). There was a non-linear regression model for the dependence of the maximum grasping mass on gender, age and the sum of thumb-index finger lengths and another non-linear regression model for the dependence of the maximum grasping diameter on the age, sum of thumb-index finger lengths and ratio of index finger to thumb length. Two regression models were useful in the optimal size design of robotic hands intending to replicate thumb-index finger grasping ability. This research can help to define not only a reasonable grasp mass and size for a bionic robotic hand, but also the requirements for hand rehabilitation.

## Introduction

By comparing with multi-fingered dexterous hands, two-finger bionic hand has simple mechanical structure and is easy for motion planning, so it is always used in the fruit harvesting robots (Bac et al., 2017; Silwal et al., 2017). However, the work environment of the fruit harvesting robots is extremely complex, such as the fruits in a plant are difference in size, shape, posture, and position (Li et al., 2019a, b), and the existing two-finger bionic hands are difficult to meet the grasping requirements of fruit harvesting (Li et al., 2013), so the robots are still not used for practical fruit harvesting so far. With the assistance of brain and eye coordination, individuals are always considered reliable performers when able to complete the tasks of grasping, moving and releasing a target fruit by using only the thumb and index finger, and the overall performance of this stable manipulation system is reasonable. The human hand is a powerful multifunctional tool, and exploration of its capabilities helps researchers to define a reasonable grasp mass and size for a bionic robotic hand, intending to replicate its abilities (Feix et al., 2014; Chen et al., 2019). From the viewpoint of ergonomics, fruit harvesting robot designers need to understand the cooperative grasping capabilities of the human thumb-index finger and the quantitative correlation between finger-length and grasping capabilities for newly designed two-finger bionic hands so as to improve their grasping performance (Yussof and Ohka, 2012; Wang and Ahn, 2017).

Studies highlighting the human thumb-index finger grasping behaviors have been published during the last decade. Some researchers revealed that the grasp stability during manipulation was primarily affected by the object weight, the relative curvature and friction force between the fingertips and object surface, and the distance between two contact points when an object was pinched by the thumb-index finger (Li et al., 2013; Luciw et al., 2014). Biegstraaten et al. (2006) concluded that the reaching and lifting movements were quite independent when an object was grasped with a precision grip (Biegstraaten et al., 2006). Vigouroux et al. (2011) proposed that when the human thumb-index finger grasped objects having different widths then the finger joint postures, muscle force and grip force varied significantly according to the object width, and an interesting result was that the muscle force/grip force ratios of the major flexor muscles remain particularly stable with respect to the width, whereas other muscle ratios differed widely. Furthermore, various research studies have also been carried out on human five-finger grasping capabilities (Vigouroux et al., 2011). Eksioglu (2004) demonstrated that the optimum grip span relative to an individual’s hand anthropometry was about 2 cm shorter than his modified thumb crotch length based on the judgment criteria of maximum voluntary isometric grip force, muscular activity and subjective rating. Seo and Armstrong (2008) illustrated that when cylindrical handles were grasped in a power grip posture, the ratio of the handle diameter to hand length can explain 62%, 57%, and 71% of the variances in grip force, normal force and contact area, respectively, Seo and Armstrong (2008). Li et al. (2010) anticipated that the hand circumference, among several anthropometric parameters such as height, weight, wrist, and forearm, lengths of hand and palm, had the strongest correlation with the maximal grip strength. Bansode et al. (2014) revealed that the dominant hand’s grip strength in males and females had significant positive correlation with the age, height, weight and body mass index and the span of the dominant hand, while it had no obvious correlation with the waist circumference and waist to hip ratio. Feix et al. (2014) found that the optimum grasping capability of the human hand was less than 500 g in terms of mass, and the width of the object at the grasp location was less than 7 cm.

In summary, significant progress has been made in this field. Nevertheless, less attention has been paid to the cooperative grasping capability of human thumb and index finger and its influencing factors. This means there is a technical gap for the ergonomic design of robotic hands intending to replicate the ability of the human hand. Therefore, on the basis of the existing literature data, we carried out studies in an effort to investigate the effect of human body characteristics (e.g., age, gender, stature, hand-used, sum of thumb and index finger lengths and ratio of index finger to thumb length) on the thumb-index finger grasping capabilities, namely, the maximum grasping mass and diameter of the thumb-index finger using a multiple non-linear regression analysis method.

## Materials and Methods

### Material

**FIGURE 1:**
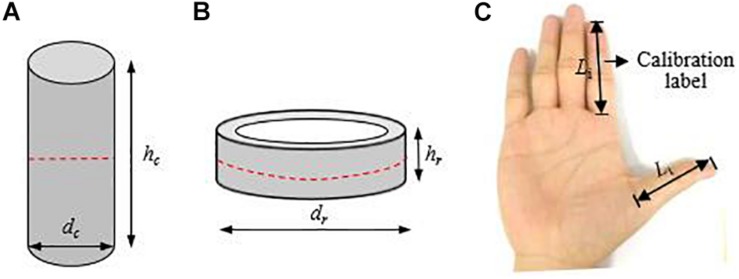
Grasped object sizes and hand sizes: **(A)** solid cylinder, where *d*_*c*_ and *h*_*c*_ denote the diameter and height, **(B)** ring, where *d*_r_ and *h*_r_denote the external diameter and height, **(C)** hand sizes, *L*_i_ – index finger length, *L*_t_ – thumb length.

To investigate the cooperative grasp capabilities of human thumb-index finger, 20 different masses of solid cylinders and 15 different external diameters of rings were manufactured as grasped objects in August 2017. The solid cylinders with a diameter *d*_c_ of 40 mm were made of C45 carbon steel having the following characteristics: density of 7.85 g/cm^3^ and surface roughness *R*_a_ = 0.1 μm (Figure 1A). The rings with a height *h*_r_ of 40 mm were made of acrylic and had a density of 1.2 g/cm^3^ and a surface roughness *R*_a_ = 0.05 μm (Figure 1B). The operative parameters of the solid cylinders and rings such as their heights, diameters and masses are listed in Table 1. The solid cylinders were used to study the effects of human body characteristics on the maximum mass of objects that can be grasped with the thumb-index finger, while the rings were used to study the effects of human body characteristics on the maximum diameter of objects that can be grasped with the thumb-index finger. Multi-factor grasp-release tests were performed within 72 h at room temperature (24 ± 1°C, 50–55% RH).

**TABLE 1:** Geometric characteristics of grasped objects.

**No.**	**Solid cylinder**			**Ring**		
	**Height *h*_c_*/_*mm*_***	**Diameter *d*_c_*/_*mm*_***	**Mass *m*_c_*/g***	**Height *h*_r_*/_*mm*_***	**Diameter *d*_r_*/_*mm*_***	**Mass *m*_r_*/g***
1	20	40	197.2	40	30	12.2
2	30	40	295.8	40	40	16.7
3	40	40	394.4	40	50	21.3
4	50	40	493.0	40	60	25.8
5	60	40	591.6	40	70	30.3
6	70	40	690.2	40	80	34.8
7	80	40	788.8	40	90	39.3
8	90	40	887.4	40	100	43.9
9	100	40	986.0	40	110	48.4
10	110	40	1084.6	40	120	52.9
11	120	40	1183.2	40	130	57.4
12	130	40	1281.7	40	140	61.9
13	140	40	1380.3	40	150	66.5
14	150	40	1478.9	40	160	71.0
15	200	40	1971.9	40	170	75.5
16	250	40	2464.9			
17	500	40	4929.8			
18	750	40	7394.7			
19	1000	40	9859.6			
20	1250	40	12324.5			

### Participants

A total of 108 volunteers (54 males and 54 females) were recruited from Henan Polytechnic University, *HPU* Kindergarten and *Hexiang* Primary School to participate in the grasp-release tests in this study. Their characteristics were as follows {mean [standard deviation (SD)]}: age, 11.9 (6.8) years; height, 141.5 (23.8) cm; thumb length, 41.9 (7.7) mm and index-finger length, 56.6 (10.0) mm. All the contributors were provided with a detailed description of the objectives and requirements of the experiment and then written informed consent was obtained from the participants over the age of 16 and from the parents of the participants under the age of 16. All the volunteers were right-handed, with normal hearing and corrected-to-normal vision, and had no history of injuries to their hands, mental illness or physical disabilities. This study was carried out in accordance with the principles of the Basel Declaration and recommendations of the Establishment of Institutional Ethics Committees in China.

### Procedures

The participants washed their hands with soap and water and dried them with a towel about 5 min before tests. The stature of each participant was measured using a folding ruler to an accuracy of 1 mm. Each participant was facilitated with a calibration label patching on his hands, and his thumb and index finger were opened to take a photo with a digital camera (Canon IXUS 95IS) from the top of the palm (Figure 1C). The captured images were transmitted into the computer and then processed by the Digimizer Version 4.2.6.0 to extract the lengths of the two fingers. The lengths of the thumb and index finger were measured by the distance from proximal flexion crease of the finger to the tip of the respective finger (Figure 1C), which was in agreement with Kanchan and Krishan (2011), Ishak et al. (2012) and Jee et al. (2015). Subsequently, the grasped objects were placed on a table, and each participant was directed to sit in an office chair in front of the table with the right upper arm parallel to the trunk, the elbow resting on his/her right thigh and the forearm extended anteriorly. The participant was asked to lift and move an object from one position to another using the thumb and index finger of the right hand naturally. 15 s later the participant moved the object back using the thumb and index finger of the left hand. During grasping, the middle finger, ring finger, little finger and palm could not touch the object.

**FIGURE 2:**
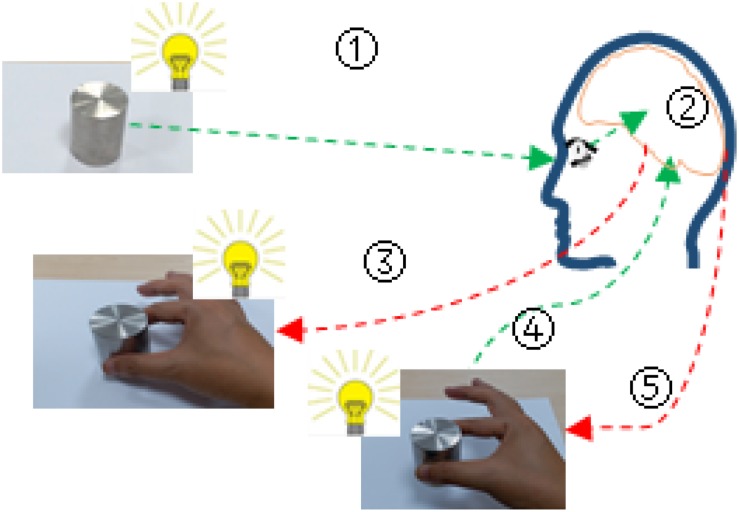
Cooperative grasping behavior of human thumb and index finger.

The cooperative grasping process of human thumb and index finger was a hand-brain-eye coordination behavior and can be divided into five steps (Figure 2). 1st step: location and sensing an object by vision system with guide of brain; 2nd step: the brain processes information obtained from vision system and makes a strategic decision (e.g., pre-grasp type, grasp force, and grasp position) for stable grasping; 3rd step: the brain commands hand to reach and grasp the object; 4th step: the tactile sensory information was feedbacked to the brain for further decision-making and if necessary the posture and force of grasp will be adjusted with the command of brain; 5th step: the hand grasps the object stably and moves it into another position.

In this experiment, the grasped objects were solid cylinders of various masses (Figure 1A) and rings of various sizes (Figure 1B). Each participant grasped the solid cylinders based on its mass from light to heavy in order and then grasped the rings based on their external diameter from small to large in order. The maximum grasping mass of the human thumb-index finger indicated the maximum mass of objects that can be grasped with the thumb-index finger. The maximum grasping diameters of the human thumb-index finger indicated the maximum diameter of objects that can be grasped with the thumb-index finger. After each grasping task, the grasping result, namely, success or failure, was recorded carefully by a skilled observer. A grasp trial was characterized as successful if the grasp-release process was stable and no relative slip occurred between the index finger, thumb and object; otherwise, it was characterized as a failed trial. In total, there were 7560 grasp trials (108 volunteers × 2 hands × 20 solid cylinders + 108 volunteers × 2 hands × 15 rings) in the experiment.

### Non-linear Regression Analysis

In this study, a non-linear regression analysis method is used to find two potential mathematical models of the relationships between the dependent variables (namely, the maximum grasping mass, and diameter of the thumb-index finger) and a set of independent variables (e.g., age, gender, hand-used, and sum of thumb-index finger lengths, ratio of index finger to thumb length). Because human thumb and index finger are co-existed and their lengths exist multicollinearity, two relative independent parameters: sum of thumb and index finger lengths and ratio of index finger to thumb length were selected to characterize the thumb and index finger lengths in the regression analysis. In consideration of the strong correlation between the stature and finger-length sum which was anticipated by Abdel-Malek et al. (1990), the finger-length sum was considered in the following regression analysis but the stature was not considered. Human body characteristics such as age and sum of thumb-index finger lengths were regarded as the original independent variables, and the maximum grasping mass and diameter of the thumb-index finger were set as the first and second dependent variables, respectively. After the experiments, the linear (e.g., *y* = *kx*) and non-linear (e.g., *y* = *kx*^2^, *y* = *k*ln*x*) functional relationships between age and maximum grasping mass, between the sum of thumb-index finger lengths and maximum grasping mass, between age and maximum grasping diameter, and between the sum of thumb-index finger lengths and maximum grasping diameter, were estimated using the “curve estimation” of IBM SPSS Statistics 24.0 (version 24.0, IBM Corporation, United States) and then compared in order to select an optimal functional relationship between the two variables based on the adjusted coefficient of determination *R*^2^. A larger *R*^2^ indicated that the corresponding function relationship is more suitable for fitting the experimental data between the two variables. The constant was not included in each regression equation.

After the optimal functions between the original quantitative independent variables and the dependent variables were obtained, each non-linear function was regarded as a new independent variable to be used in the following multiple linear regression analysis and the significance level was set at 0.05. Because gender and the hand-used were categorical variables, before linear regression analysis, the two levels of gender, namely, male, and female, were coded as “0” and “1,” respectively, and the two levels of the hand-used, namely, left hand and right hand, were also coded as “0” and “1,” respectively. Finally, a multiple linear regression analysis was used to construct two potential mathematical models. The constant was not included in each regression model. The goodness-of-fit test was used to measure how well the observed data correspond to each regression model, the *F*-test was used to test the overall significance of each regression model, and the *t*-test was used to determine whether an independent variable had a statistically significant effect on the dependent variable in each model.

## Results and Discussion

### Descriptions of Experimental Results

**FIGURE 3:**
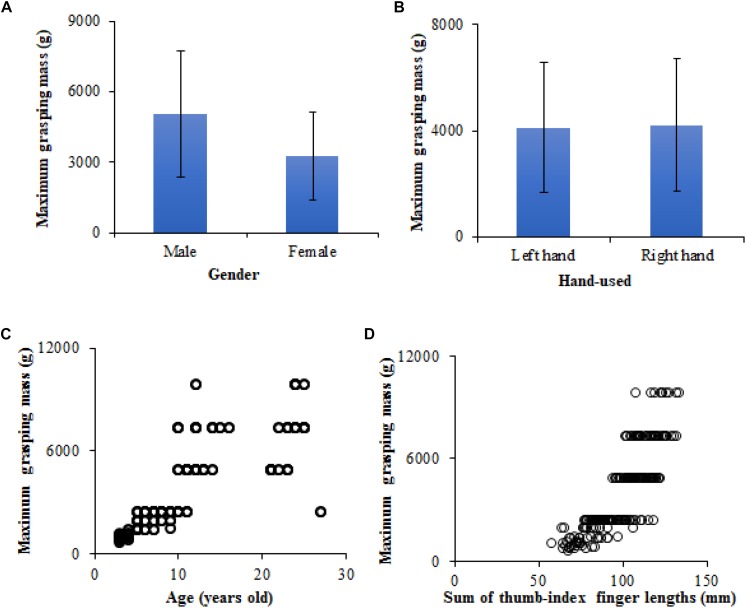
Maximum grasping masses of the human thumb-index finger under different human body characteristics conditions: **(A)** Relationship between gender and maximum grasping mass (Mean ± Standard Deviation), **(B)** relationship between hand-used and maximum grasping mass (Mean ± Standard Deviation), **(C)** relationship between age and maximum grasping mass, **(D)** relationship between sum of thumb-index finger lengths and maximum grasping mass.

Figure 3 shows the maximum grasping masses of the human thumb-index finger under different human body characteristics conditions (e.g., gender, hand-used, age, and sum of thumb-index finger lengths). In this study, the maximum grasping masses of the thumb-index finger of participants ranged from 690.2 to 9859.6 g. The maximum grasping masses of the thumb-index finger of male participants were 5057.6 ± 2695.6 g (Mean ± Standard Deviation), significantly higher than those of female participants 3265.5 ± 1853.5 g (Figure 3A). However, there was no significant difference in the maximum grasping masses of the thumb-index finger in the left and right hands of participants; the maximum grasping masses of the thumb-index finger of the left hand were 4102.7 ± 2449.4 g, slightly lower than those of the right hand 4220.5 ± 2513.1 g (Figure 3B). In this study, the age of participants ranged from 3∼27 years old and the sum of the thumb and index finger lengths ranged from 56.9 to 132.6 mm. Obviously, the maximum grasping masses of the thumb-index finger of participants had a non-linear (e.g., quadratic function, logarithmic function) increased trend with increasing age and the sum of the thumb and index finger lengths (Figures 3C,D).

**FIGURE 4:**
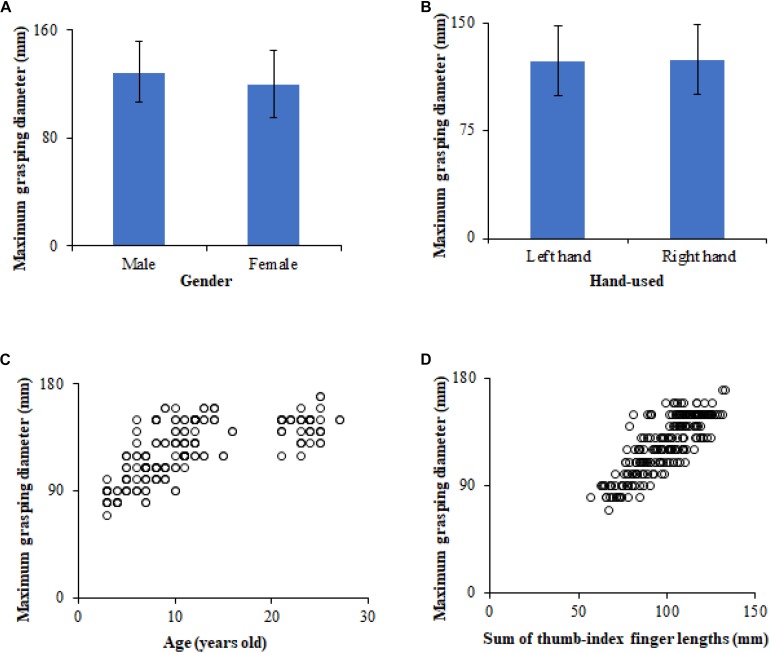
Maximum grasping diameters of the human thumb-index finger under different human body characteristics conditions: **(A)** Relationship between gender and maximum grasping diameter (Mean ± Standard Deviation), **(B)** relationship between hand-used and maximum grasping diameter (Mean ± Standard Deviation), **(C)** relationship between age and maximum grasping diameter, **(D)** relationship between sum of thumb-index finger lengths and maximum grasping diameter.

Figure 4 shows the maximum grasping diameters of the human thumb-index finger under different human body characteristics conditions (e.g., gender, hand-used, age, and sum of thumb-index finger lengths). In this study, the maximum grasping diameters of the thumb-index finger of participants ranged from 70 to 170 mm. The maximum grasping diameters of the thumb-index finger of male participants were 129.0 ± 22.2 mm, slightly larger than those of female participants 119.9 ± 25.2 mm (Figure 4A). The maximum grasping diameters of the thumb-index finger of the left hand were 124.0 ± 24.1 mm, almost equal to that of the right hand (Figure 4B). Similar to Figures 3C,D, the maximum grasping diameters of the thumb-index finger of participants also showed a non-linear (e.g., quadratic function, logarithmic function) increased trend with increasing age and the sum of the thumb and index finger lengths (Figures 4C,D).

Table 2 lists the adjusted coefficients of determination of the linear and non-linear regression models between the quantitative independent and dependent variables. By comparing the adjusted coefficients of determination of three types of functions, the linear function provided the optimal functional relationship between age and maximum grasping mass, and between sum of thumb-index finger lengths and maximum grasping diameter; the quadratic function yielded the optimal functional relationships between sum of thumb-index finger lengths and maximum grasping mass; and the logarithmic function provided the optimal functional relationship between age and maximum grasping diameter. These results were used in the following regression analysis.

**TABLE 2:** Adjusted coefficients of determination of regression models between independent and dependent variables.

**Function**	***M*_max_ = *f(A)***	***M*_max_ = *f(L_0_)***	***D*_max_ = *f*(*A*)**	***D*_max_ = *f(L*_0_)**
*y* = *k x*	0.89	0.84	0.84	0.99
*y* = *k x*^2^	0.72	0.91	0.56	0.96
*y* = *k* ln *x*	0.88	0.77	0.97	0.98

**M*_*max*_-maximum grasping mass; *D*_*max*_-maximum grasping diameter; *A*-age; *L*_0_-sum of thumb and index finger lengths.*

### Factors Affecting the Maximum Grasping Mass of Human Thumb-Index Finger

The non-linear regression model for the dependence of the maximum grasping mass on gender, age and sum of thumb-index finger lengths is presented in Eq. 1. The adjusted coefficient of determination, which is denoted as *R*^2^, was 0.97, which suggests that the model fitted the data well and indicates that this model can explain 97% of the variance in the maximum grasping mass that was predicted by the gender, age and sum of thumb-index finger lengths. It was concluded from the *F-*test that the overall fit was significant (*P* < 0.05). *t-*tests demonstrated that the maximum mass of the objects that the participants could grasp using the thumb-index finger depended on gender, age and the sum of thumb-index finger lengths (*P* < 0.05), but not on the hand-used and ratio of index finger to thumb length (*P* > 0.05).

(1) $$M_{\text{max}}=\,127.1\times A\,+\,0.32\times L_{\text{o}}^{2}\,-1070.5\times G$$

where *M*_max_ – maximum grasping mass, g; *G* – gender; *A* – age of participants, years; and *L*_o_ – finger-length sum, mm.

The age of participants ranged from 3∼27 years old, which is at the stage of growth and development of human muscle (Lexell et al., 1992), so the age showed a positive significant effect on the maximum grasping mass of the human thumb-index finger. The sum of thumb and index finger lengths was positively related to the maximum grasping mass of the human thumb-index finger, the reason being that participants with big hands have long fingers and tend to have high muscular strength (Seo and Armstrong, 2008). The values *G* = 0 or 1, namely, male or female, were substituted into Eq. 1 to describe the maximum grasping masses of the participants. The difference in the maximum grasping masses of the thumb-index finger of males and females was 1070.5 g. Similar research results demonstrated that the grip strength of males was significantly higher than that of females (Puh, 2009) and the hand length had a significant effect on the grasp strength of human five-fingers (Li et al., 2010). The significant relationship between gender and the maximum grasping mass of two fingers can be attributed to the maximal voluntary contraction force of males being always greater than that of females of similar height (Shurrab et al., 2017). Therefore, increasing the maximal voluntary contraction force can improve the maximum grasping mass of human two-fingers. The grip strength is a similar parameter to the maximum grasping mass for measuring the grasping capacity of human fingers. These findings illustrated that the finger-length sum and the maximal voluntary contraction force would jointly affect the maximum grasping mass of thumb-index finger, which suggested that the two factors should be considered together for improving the maximum grasping mass of robotic hands during ergonomic design.

### Factors Affecting the Maximum Grasping Diameter of Human Thumb-Index Finger

The non-linear regression model for the dependence of the maximum grasping diameter on the age, sum of thumb-index finger lengths and ratio of index finger to thumb length is presented in Eq. 2. The adjusted coefficient of determination, which is denoted as *R*^2^, was 0.99, which demonstrates that the model fitted the data well and this model can explain 99% of the variance in the maximum grasping diameter that was predicted by the age, sum of thumb-index finger lengths and ratio of index finger to thumb length. It was concluded from the *F-*test that the overall fit was significant (*P* < 0.05). *t-*tests showed that the maximum diameter of the objects that the participants could grasp using the thumb-index finger depended on the age, sum of the thumb-index finger lengths and ratio of index finger to thumb length (*P* < 0.05), but not on the gender and hand-used (*P* > 0.05).

(2) $$D_{\text{max}}=6.58\times L\,n\,\left(\text{Age}\right)+0.98\times L_{\text{o}}\,+9.67\,\,L_{\text{r}}$$

where *D*_max_ – maximum grasping diameter, mm; *L*_o_ – sum of thumb-index finger lengths, mm; *L*_r_ – ratio of index finger to thumb length.

The sum of the thumb and index finger lengths ranged from 56.9 to 132.6 mm and the ratio of index finger to thumb length ranged from 1.09 to 1.65. The sum of the thumb and index finger lengths was positively proportional to the maximum grasping diameter. The longer the sum of the thumb-index finger lengths, the larger the span between two fingertips; hence, the larger the maximum grasping diameter of the participants using the thumb-index finger. When the sum of the thumb and index finger lengths increased by 1 mm, the maximum grasping diameter of thumb-index finger increased by 0.98 mm. When the ratio of index finger to thumb length increased by 0.01, the maximum grasping diameter of thumb-index finger increased by 0.0967 mm. The ratio of index finger to thumb length was positively related to the maximum grasping diameter, which indicated that the combination of a short thumb and a long index-finger would increase the maximum grasping diameter of the thumb-index finger. The main reason is that as an object is grasped by two fingers, especially with the power-grasp type, the short thumb easily serves as a supporting point to match the long index finger in enveloping the object contour to form a force closure plane. The short thumb is not easy to be constrained by the object shape and a force-closure stable grasp can be achieved in the contact plane based on the grasp stability criterion that were reported by Li et al. (2013). There is little information on this topic in the literature.

## Discussion

The Eq. 1 in see section “Factors Affecting the Maximum Grasping Mass of Human Thumb-Index Finger” quantitatively described the relationship between the sum of thumb and index finger lengths and the maximum grasping mass. When developing a two-finger bionic robotic hand, if the masses of potential target objects are given, an optimal length design of robotic thumb and index finger can be deduced using the Eq. 1 and an additional condition: the average ratio of index finger to thumb length is 1.36. Similarly, the Eq. 2 in Section “Factors Affecting the Maximum Grasping Diameter of Human Thumb-Index Finger” quantitatively described the relationship between the sum of thumb and index finger lengths, the ratio of index finger to thumb length and the maximum grasping diameter. When developing a two-finger bionic robotic hand, if the diameters of potential target objects are given, a suitable length design of robotic thumb and index finger can be deduced using the Eq. 2. Hence, the two non-linear regression models were useful in the optimal size design of robotic hands intending to replicate thumb-index finger grasping ability. When manipulating a novel object, sensory feedback provides us with information about its physical properties such as mass and then the brain is thought to select the most appropriate model maintained in our central nervous system for the current task (Lemon et al., 1995; Davidson and Wolpert, 2004). According to the maximum grasping mass set of thumb-index finger, a deep learning algorithm can be developed to justify whether some objects in an unstructured working environment meet the grasping requirements of bionic robotic hands. Furthermore, if there are some irregular objects (e.g., mug) in the unstructured environment, the maximum grasping diameter set of thumb-index finger can be used to make grasp planning algorithms for selecting the optimal grasping locations on an irregular object surface for a bionic robotic hand. Additionally, many time-varying problems always exist in the kinematic control problems of robotic fingers and the varying-parameter convergent differential neural network would be an efficient and accurate method for solving this grasping planning problem (Zhang et al., 2018a, b).

## Conclusion

In this study, the maximum cooperative grasping mass and diameter of the human thumb and index finger in a wide range of unstructured tasks were investigated. The age of participants ranged from 3∼27 years old and the sum of their thumb and index finger lengths ranged from 56.9 to 132.6 mm. The results showed that the maximum grasping masses and diameters of the participants’ thumb-index finger ranged from 690.2 to 9859.6 g and 70 to 170 mm. The maximum grasping mass of the participants’ thumb-index finger depended on gender, age and the sum of thumb-index finger lengths (*P* < 0.05), but not on the hand-used and ratio of index finger to thumb length (*P* > 0.05). The maximum grasping diameter of the participants’ thumb-index finger depended on the age, sum of the thumb-index finger lengths and ratio of index finger to thumb length (*P* < 0.05), but not on the gender and hand-used (*P* > 0.05).

There was a non-linear regression model for the dependence of the maximum grasping mass on gender, age and the sum of thumb-index finger lengths and another non-linear regression model for the dependence of the maximum grasping diameter on the age, sum of thumb-index finger lengths and ratio of index finger to thumb length. Two regression models were useful in the optimal size design of robotic hands intending to replicate thumb-index finger grasping ability. This research can help to define not only a reasonable grasp mass and size for a bionic robotic hand, but also the requirements for hand rehabilitation.

## Data Availability Statement

All datasets generated for this study are included in the article/supplementary material.

## Author Contributions

XC and ZL designed and performed the experiments and wrote the manuscript. YW, JL, and DZ reviewed and supervised the work.

## Conflict of Interest

The authors declare that the research was conducted in the absence of any commercial or financial relationships that could be construed as a potential conflict of interest.
